# New York Odontological Society

**Published:** 1890-04

**Authors:** 

**Affiliations:** New York Odontological Society


### Reports of Society Meetings.

### NEW YORK ODONTOLOGICAL SOCIETY.

THE New York Odontological Society held its regular monthly meeting, Tuesday evening, January 21, 1890, in the New York Academy of Medicine, No. 12 West Thirty-first Street.

The president, Dr. J. Morgan Howe, in the chair.

### INCIDENTS OF OFFICE PRACTICE.

*Dr. Geo. S. Allan.*—Mr. President, I have here an amalgam carrier that I would like to show the gentlemen present. I do not know of any instrument in the market that will meet the good points of this one. A slight pressure opens the nibs to receive the amalgam, and when it is carried to the cavity the nibs are pressed apart and the amalgam placed wherever desirable with the other hand. There is no danger of dropping the amalgam on the way from the desk to the cavity, and the instrument works well in every way. The spring is very light, just enough to hold the amalgam without pinching it, and the beaks are a little serrated inside, so that the amalgam does not drop off when the nibs are separated.

I have long wanted a syringe for general work in the mouth that would be free from many of the objectionable features of those we ordinarily purchase. The rubber syringe never works well after it has been used a short time; the piston soon wears in the barrel and the suction becomes imperfect. This one, which I now exhibit, is a glass syringe, and it holds about the requisite quantity. When it is being filled there is no loss whatever; no air follows the fluid; and it works smoothly as only a syringe with a glass or metal barrel will. I have two or three nozzles here; two for reaching into the antrum. One of these is something on the principle of Dr. Farrar's spray syringe. It throws the fluid in all directions, and there is no difficulty in treating diseases of the antrum with any medicine that it is desirable to use. Then I have a third nozzle for ordinary work in the mouth, for pus pockets, or cavities where teeth have been extracted and there are indications of blood-poisoning. Take it as a whole, it is one of the handiest little instruments that I have ever had for such purposes.

When syringes are used only occasionally and are then laid away, the plunger soon becomes dry, and has to be soaked before it will work. That difficulty can be obviated by taking off the nozzles and placing the syringe in a glass jar or bottle with a little water in it. They are then ready for use at any moment. The Farrar syringe is a most useful little instrument, but I have heard complaints about its becoming contrary and refusing to work; but if it be put into a bottle after using, as I have suggested, it will always be ready to do its work.

*A Voice.*—Where can this syringe be purchased.

*Dr. Allan.*—This I bought of a surgical instrument-maker on Sixth Avenue, just below Thirty-fifth Street, east side, next to Cherry's trunk store. He made these nozzles to order.

With the consent of the president, I would like to read from the *Dental Review*, of January 15, a few paragraphs from an article headed "Ethics, Professional and Otherwise," and signed "Nemo." This article attempts, in a very *naïve* way, the defence, as I take it, of the Dental Trade Association, and indirectly raps the profession over the knuckles for intermeddling with the business of others. After I have read the paragraphs I will tell you why I read them.

"Much has been said and written in regard to the impropriety of dental patents secured by dentists, and, by sale or otherwise, made a source of revenue to the inventor. It would seem to be not difficult to defend the rights of the individual in this matter. If he has voluntarily become a member of a dental society, he is in honor bound to abide by its rules or to withdraw from its membership; and, on the other hand, members of the society may properly refuse to recognize professionally those they believe to be acting in a manner adverse to the interests of the profession. But when dentists go beyond this, and practically claim the right to establish a code of ethics for those engaged in an entirely different occupation, they invite the like interference with, or at least criticism of, their own acts. There has been much sharp denunciation of dealers in dental goods because of an association formed by them, also of certain manufacturers, because of their purchasing patents, and in various ways so managing their business as to make it most profitable to themselves. While trusts are multiplying and are made for the acknowledged purpose of preventing competition, controlling production, and advancing prices, it is gratifying to know that this Dental Trade Association has no power to prevent competition, limit production, or fix prices; and without such power it is certainly not much to be feared by the public.

"Manufacturers and dealers in goods of all kinds do business for profit in hard cash, and the struggle for success is always sharp and often unsuccessful. Much work in every branch of business is done without profit, and yet this part cannot be rejected, as it is essential to retain the other. The grocer who should refuse to sell sugar because there is no profit in it would soon find his customers going elsewhere for other goods besides sugar."

Now I want to say that there has seldom been put in a few lines a stronger argument in favor of independent journalism than that contained here. It is because dentists acknowledge openly, and assert openly, that this Trade Association is governed by different rules and a different code of ethics from others, that they want to have their journals independent. They do not want their journals dictated to by an association or a business concern that is antagonistic to the principles that they wish to adopt, and that they do adopt in their daily professional life. It is for the interest, as this writer plainly says, of the Trade Association to deal in patents, to purchase patents, and to obtain the control of patents, and this for the sake of making money. Now, the ground taken by the dental profession is that we dentists ought not to patent articles ourselves or allow a product of our brains to be made merchandise of by others. I do not think there is any question about that; therefore this plain, unvarnished statement of a defender of the Trade Association, I think, calls for a slight notice. He says there that the association has not the power to prevent competition, limit production, or fix prices; and that without such power it is not much to be feared by the public. I do not pretend to know what the rules that govern that association are, but I do know this as a fact: that no dental dealer or manufacturer can start in business anywhere in these United States and be successful unless he joins that association. That is a simple fact which men here present know to be such; and that certainly indicates that the profession, as a whole, are right in their position of antagonizing this association, because it does not allow of free trade in the necessities which we daily require in practice.

*Dr. S. E. Davenport.*—It may not be out of place at this time for me to give favorable testimony concerning Mr. R. S. Williams's crystalloid gold, No. 3, which I have been using several months.

I am surprised, when thinking the matter over, that I have come to value it so highly, for I was not at all pleased with the previous numbers of the same gold, although I gave them, as I thought, a fair trial. The No. 3 can be placed in a cavity without crumbling, is easily pressed into an undercut or other inequality, and it seldom tips up when starting a filling, though the unevenness of the cavity be very slight. This gold never seems harsh; on the contrary, it possesses a lead-like quality fully equal to the combination of tin and gold, is sufficiently cohesive without annealing for all ordinary work, and even when heated to a red heat to anneal for large contours it does not become stiff. While experimenting with this gold I have been led to use it from the foundation, in a number of large fillings, where considerable masses of gold were necessary for contours and new biting surfaces, and the result has been very satisfactory. The surfaces are very hard, about the same, I should say, as crystal gold, and I expect those fillings to be durable, if the cavities have been properly prepared. While the question of time is less important than many others, it yet means much to all busy men, and therefore it is only fair to state that with the No. 3 crystalloid a filling can probably be made more rapidly than with any other cohesive gold.

Following the method which was so ably introduced last year before this Society by Dr. Dwight M. Clapp, of Boston, namely, the combination of amalgam and gold in the same cavity at one sitting, I have found that No. 4 crystalloid gold—which is, I think, prepared especially for work of that character—is in my hands superior to Steurer's gold, which Dr. Clapp was using at that time. It is not so crumbly, the parts being held together by a layer of foil on each side, and it can therefore be carried to place and condensed without breaking to pieces; and that I never could do with Steurer's gold, unless the cavity was in the lower jaw, when gravitation would assist. Dr. Kingsley being present, I hope he will say a few words in explanation of his early efforts to combine gold and amalgam. He succeeded, I know, but although I have tried to find a full description of that success, the effort has been in vain.

*Dr. N. W. Kingsley.*—It does not seem to require many words. I remembered while the gentleman was speaking on the subject that I, with some timidity, brought it to the attention of the State Society the first time I ever mentioned it publicly. I say with timidity, because I knew the prejudice there was against amalgam. I remember especially one gentleman making comments upon it of this nature: he never used anything but gold, and never had any difficulty whatever in using it; any man who could not use gold successfully proved his inability to do work properly; and to fall back upon amalgam for a substitute in the way which I described proved great lack of skill. But the mills of the gods grind slowly, as do some other mills; and I knew if it was worth anything it would be accepted in time; and, perhaps, if it was not worth anything. My experience has been that, whether a thing is good or bad, sooner or later many of the profession will be found adopting it and lauding it to the skies; the professional pendulum will sooner or later swing from one extreme to the other. I never had any serious difficulty in combining gold and amalgam; but to say I never had any difficulty would not be stating a fact. I do not know of anything in dentistry that I have not had some difficulty in doing at first. I used amalgam in that way because I was forced to. I felt compelled to use something against the cervical edges of approximal cavities of molars and bicuspids where they were not easily reached, cavities where the space between the teeth was somewhat narrow. It seemed to me that if amalgam was good under any circumstances, there was no objection to putting it there; particularly as I had seen many of the best operators, when good gold fillings in such places as that had given out, repair them with amalgam; and I did not know why it would not be as well to use amalgam in the beginning. I used a small quantity of amalgam, and packed the gold against it. In some cases the gold would continue to absorb the mercury from the amalgam until the cavity was one-half or two-thirds full, but I would keep on with the work and subsequently the gold would unite.

Fillings made in that way stand. That is my experience, and I think it is also the experience of others. They are not as liable to give out along the border as fillings made entirely of gold. I do not think there is anything especial in my experience or method of doing that kind of work.

*Dr. Davenport.*—The question in my mind was not as to the advisability of combining amalgam and gold in that class of cavities, for I think the profession has grown sufficiently to recognize the value of such fillings. The point was whether Dr. Kingsley used a special gold and succeeded in getting a chemical union. I have been able to get a definite union of gold and amalgam with but two preparations of gold; those preparations are Steurer's gold and R. S. Williams's crystalloid, No. 4. Of course, if Dr. Kingsley filled only one-third to one-half the cavity with amalgam and then began with the gold, packing it into undercuts, he would not need a definite union; but, if he recollects Dr. Clapp's paper on that subject, it was stated that most of the filling, including the knuckle, was made of amalgam with only a thin capping of gold which did not extend into undercuts, but depended entirely upon its union with the amalgam.

*Dr. George A. Mills.*—Dr. Kingsley's idea of combining gold and amalgam was probably first suggested by the necessity for repairing large gold fillings. I remember performing some very extensive operations with No. 60 and No. 120 gold for one patient who was undergoing a good many constitutional changes, and these conditions manifested themselves in the teeth. He came to me in 1880 or 1881 with gold fillings on the buccal surfaces of the teeth and decay had recurred. I thought the best thing I could do was to cut out and fill with amalgam. I did so. After a year I found extensive decay on the approximate surfaces and lingual surfaces. I cut that out and filled in the same way, following right around at different times, until now I have gone entirely around the teeth. I saw the gentleman four or five weeks ago, and he spoke particularly of the manner in which I had saved his teeth. I consider it was due to the combination of metals. I have seen an immense amount of mischief done in filling teeth with amalgam, especially approximate cavities. Extensive fillings are found where, although the cavities were large in the first place, the decay dips in under the edges of the fillings. I have come to the definite conclusion, in treating those cases, that the amalgam was placed in the teeth because the dentist could not do anything better. In many cases I have cut out the fillings and filled with amalgam, restoring the contour with gold. I have come to be a believer in the general characteristics of Dr. Palmer's theory in regard to the combination of metals, and am a strong advocate of the combination of tin and gold. I have had several years experience in it, and have talked with a good many intelligent dentists who believe with me that teeth can be saved by a combination of metals when perhaps they could not be saved in any other way.

*Dr. Lord.*—Mr. President, we have Dr. Palmer, of Syracuse, here this evening.

*The President.*—We shall be pleased to hear from Dr. Palmer.

*Dr. S. B. Palmer.*—Mr. President, I think all has been said on the subject that is necessary. I remember very distinctly being present when Dr. Kingsley made his first announcement in regard to the combination of gold and amalgam. I was meeting with so much opposition at that time for having advocated the use of tin and gold in contact that I did not dare to include amalgam, and did not for several years after because of the prejudice against it. When Dr. Kingsley was about to make his announcement to the State Society, he came to me and asked what I thought of the practice he was going to introduce. I told him to go on, that the principle was right and he was safe in advocating it.

In regard to the combination of tin and gold, unless the two metals are properly mixed, as I have previously described, so that the tin and gold form an alloy which resists oxidation, I have found that the tin portion of the filling was affected by galvanic action, becoming a soft black mass. Seldom is there any decay, but an instrument can be inserted through the tin portion of the filling. No doubt others, who have used tin and gold in combination, have noticed like conditions. Experience teaches that amalgam is more reliable than tin at the cervical border as a guard material under gold. Some operators seem to have difficulty in anchoring the gold to a freshly-inserted amalgam base. Alloy for this purpose should be kept in the ingot, and be cut for each operation, and not amalgamated till after the cavity has been dried ready for filling. Amalgam thus prepared, unless there is more than the right proportion of mercury, will become firm while packing the first layers of gold, and will not cause the gold to slide upon the surface, because of the presence of free mercury. Occasionally it occurs that gold must be added to or built over amalgam previously inserted. It is very important that all surfaces which come in contact with gold be touched with mercury, so that the gold may be soldered to the amalgam; the two metals, thus united, never show the joint by wear or chemical action, because the mercury, thin as the coating is, becomes an amalgam containing gold of higher potential or finer than the body of the plug. Practically, the gold element passes over the line of union into the body of the amalgam.

Quite the reverse of this occurs when amalgam is added to amalgam, even though the potential may be nearly the same between the plugs. Of course, the joint is made perfect by the newly-inserted material being soldered to the old, but the alloy or solder which joins the two is not as fine as either other element, containing, as it does, more mercury. The result is, chemical action works upon the joint; a slight seam or hair-line is first noticed, and after a few years the pieces separate. A single thickness of gold foil, placed between the two plugs, would secure a perfect joint, the connecting element would be negative or finer than the body of the filling. Those who have made temporary plates of silver, where the backings of the teeth were fastened to the plate with soft solder, know that the teeth with the rim of solder would separate from the plate in a few months. Certainly was this the case where the tinning of the plate was done with chloride of zinc, because the tinning became an alloy of silver, zinc, tin, and lead, and was decomposed by galvanic action.

*The President.*—Dr. Merriam has sent for exhibition fourteen samples of files for finishing fillings and filing enamel edges. They are curved on the edge, and are all different. I will pass them around, so that the gentlemen may see what excellent and practical ideas Dr. Merriam has had in devising files for these purposes.

The secretary being absent, I will ask Dr. Davenport to favor us by reading the paper of the evening, by Dr. George H. McCausey, of Janesville, Wis., on "The Relations of the Tooth-Pulp to the other Tooth Tissues." (For paper see page 193.)

*The President.*—Gentlemen, you have heard Dr. McCausey's interesting paper; the subject is now open for discussion.

*Dr. Carl Heitzmann.*—Mr. President, you have been kind enough to invite me here to-night, and I feel that I am amply repaid for coming. Last year I listened to a paper by Dr. Ingersoll on this subject. Dr. Ingersoll maintained that the whole pulp is nerve tissue, and that the pulp itself is a ganglion. I have fought that theory as much as I could, and now I am really delighted that a gentleman, whom I do not know personally, takes exactly the same ground that I took last year, and have taken since I began studying the histology of the teeth, more than thirty years ago.

The gentleman exhibits a familiarity with certain minute anatomical features of the pulp which both delights and surprises me. He speaks of the delicate fibrillæ passing through the protoplasm and connecting the granules with one another, going from one cell to another, and making the whole a continuous tissue. I am sure Dr. Allan, who is present to-night, will be delighted to hear that. He denies it. But here is a gentleman, who has never studied in my laboratory, and never drew the reticulum in my laboratory, and, nevertheless, describes it as existing throughout the protoplasm which builds up the pulp tissue. After all, gentlemen, if one does not get his reward quickly, when working hard and earnestly, he does get it sooner or later. This is a reward to me, for the gentleman seems to corroborate my own statements; and, with the exception of some slight discrepancies of no importance, I can agree with what he has said. Although he does not mention Dr. Ingersoll he evidently refers to his views. He perhaps does not want to personally attack Dr. Ingersoll; but as far as I am concerned, I have no such objections. The essayist says that the basis substance of the dentine is cartilaginous. That is a slight mistake. It is a glue-yielding substance, but not cartilaginous. When boiled, it yields a cloudy liquid smelling like glue, but not viscid. He maintains that the odontoblasts, which he does not exactly nominate as such, though giving an accurate description of them, send offshoots into the dentine, and he claims that these offshoots are not nerves. We all of us know that John Tomes alluded to the possibility that the tenants of the dentinal canaliculi might be nerves. It is perfectly surprising, gentlemen, how near this excellent investigator, who is still living, approached the truth. If the essayist had used, instead of protoplasmic threads, the term that Dr. Boedecker and myself have chosen,—living matter,—he would have pretty nearly stated the facts correctly. The nerves, as such, are undoubtedly forms of living matter, in contradistinction to what Lionel Beale says of the basis substance of connective tissue being "forming material." How is it possible that an almost inert mass, such as the basis substance of connective tissue is, should be compared, as Beale does, with the most active tissue that we have in our bodies,—the nerve and muscle tissue. This mistake was demonstrated by Bastian, and I have simply upheld his theory. What we call nerve is not formed material, neither is what we call muscle; but both are living matter. If we take this ground and say that living matter pierces the dentine in all possible directions, being merely stored up in the shape of filaments that are able to conduct sensation, and that are able to supply the whole dentine with what we call the properties of life, nutrition and growth included, then we have said pretty nearly everything. Then we have asserted that the living matter, being within the canaliculi of the dentine, is in one essential point identical with the living matter building up the nerves. This is the ground taken by Dr. Boedecker and myself. We have looked a long time for the immediate connections between the dentinal fibres and the non-medullated nerves, and I can say that we have failed to see such direct connections. Some very sensible dentists assume that such a connection must exist. In cutting a tooth we know that as soon as we have reached the boundary zone—the interzonal layer of Dr. Atkinson, between the enamel and the dentine—there is a keen sensation of pain; and another point where there is marked sensibility is the neck of the tooth. How shall we explain the carrying of what we call sensation from far up at the boundary of the dentine to the tips of the nerves, unless there is something living within the dentine? Therefore we have to admit that a connection exists between the dentinal fibres and the non-medullated nerve fibres; but this connection is not a direct one. In other words, the dentinal fibres do not directly inosculate with the nerve fibres, but there is an indirect connection existing. That means that this very reticulum, which the essayist described, and which unquestionably exists, furnishes the material by means of which the dentinal fibres are connected indirectly with the nerves. If we say that this reticulum, which the gentleman calls the protoplasmic reticulum,—we prefer the term living reticulum,—if we say that from the top of the crown down to the pulp-chamber there is such a continuity of a living reticulum, we have said pretty nearly everything that we understand of the dentine and its marked sensibility. Besides, it explains a fact which Dr. Boedecker first demonstrated, —that the points of greatest sensibility are at the interzonal layer between the dentine and the enamel, and at the neck of the tooth between the dentine and the cement; for at those points there is more living matter and a coarser reticulum than is found throughout the rest of the dentine or of the cementum.

I can say that, with a few exceptions, I concur with the gentleman who wrote this paper. Evidently he has really studied with the microscope, in contradistinction to Dr. Ingersoll, who admits that he did not; and in contradistinction to some other men who claim to know something about the microscope and who do not know even the elementary principles of microscopy. This gentleman truly says it is bad practice to mount sections of teeth in Canada balsam. He is right. We have demonstrated years ago that it is a blunder to mount specimens in Canada balsam. I heartily and cordially agree with the essayist in every essential point.

*Dr. Allan.*—The old Scotch verdict, not proven, which the author of that paper alludes to, certainly is the verdict not only of the writer of the paper, but is the verdict of the histologists of the present day, so far as any direct relationship can be shown between the contents of the dentinal tubuli and the pulp tissue. But it is not necessary that the contents of the dentinal tubuli should be nerve tissue in order to convey sensation. Protoplasm itself conveys sensation most decidedly. Any one who has studied under the microscope the lower forms of animal life, such as the protistæ

and the monads, will have direct evidence that protoplasm, long before there has been any differentiation that can be demonstrated by any microscope, possesses what we would call sensatory power. In other words, it seems to manifest life to a certain extent, in action, motion, and direction in avoiding obstacles, and in many other ways; therefore it is not at all necessary to suppose that these contents of the dentinal tubuli are directly or indirectly nerve tissue, because in some way they do convey sensation, for that sensation is a property of protoplasm is a truism that cannot be questioned.

In regard to the reticulum, it is barely possible that, as Dr. Heitzmann claims, this paper upholds his theory. It is a theory that I have often invited Dr. Heitzmann to put in my hands specimens to prove and in which I do not believe.

*Dr. George Evans.*—I have been in Dr. Heitzmann's laboratory, and have studied with him. The reticulum that has been discussed here this evening I have seen distinctly, both in the dentine and in the enamel, as represented by Dr. Boedecker and Dr. Heitzmann.

*Dr. Allan.*—I know of several besides myself who are exceedingly interested in this subject, men of character and of acknowledged technique; histologists, who are very anxious to obtain specimens to prove whether this marvellous reticulum exists or not. If any gentleman here can put me in the way of obtaining such a slide I will be very much obliged to him, and will put it to a good use.

*Dr. Atkinson.*—In my acquaintance with scientific men I have met with no one who so entirely captivated me in the demonstration of what he really sees in the microscope as Dr. Heitzmann.

The point of interest in this paper is to find out what function is. I think we are nearing it. I was inspired by some pupils that I had to undertake to show that embryonal tissue made its first appearance in a neural mass, so that it included all possibilities of function within it, and the differentiation of that material afterwards gave us the lay-out of the five tissues that we are so well acquainted with. I think that little key will unlock the whole difficulty of the conductivity of protoplasm, and the conductivity of the same thing when it is in shape of a nerve mass called neurine, which is nothing less than protoplasm. I think it merely a matter of difference in verbiage.

Adjourned.

S. E. Davenport, D.D.S., M.D.S.,
*Editor New York Odontological Society.*

